# Admission and Mortality Patterns in Intensive Care Delivery at Enugu State University of Science and Technology Teaching Hospital: A Three-Year Retrospective Study

**DOI:** 10.7759/cureus.27195

**Published:** 2022-07-24

**Authors:** Jonathan Eya, Mazpa Ejikem, Chidubem Ogamba

**Affiliations:** 1 Anesthesiology, Enugu State University of Science and Technology Teaching Hospital, Enugu, NGA; 2 Medicine and Surgery, University of Uyo, Uyo, NGA

**Keywords:** mortality, mortality patterns, icu, icu mortality rate, admission patterns, intensive care unit

## Abstract

Background

The intensive care unit (ICU) provides critical care to high-risk patients to prevent morbidity and mortality. This requires closer monitoring and better management than the care provided to patients in normal admission wards and non-critical care units. Mortality rates in ICUs in developing countries are remarkably high compared to rates in more developed countries. Evaluating outcomes of treatment is a way to improve the quality of care. Therefore, this study was conducted to review the pattern of admission and outcome in the ICU of Enugu State University of Science and Technology Teaching Hospital (ESUT-TH).

Methodology

This study was a three-year retrospective, descriptive review of all patients admitted to the ICU of ESUT-TH between January 1, 2019, and December 21, 2021. Data were collected from admissions and discharge registers of the ICU ward. Data were analyzed and expressed as frequencies and percentages. Categorical parameters were compared using the chi-squared test, and the significance level was set at p < 0.05.

Results

A total of 179 patients were admitted in the three-year period. Of them, 49.2% were postoperative patients while 21.2% were admitted from the accident and emergency unit. There were a total of 74 (41.3%) medical cases and 81 (45.3%) surgical cases, and the rest were unspecified. Among surgical cases, 19% were from the general surgery department followed by obstetrics and gynecology (18.4%) and neurosurgery (16.8%). Cerebrovascular accidents and traumatic brain injury were the most common specific diagnoses recorded among ICU admitted patients. The most common reason for admission was close monitoring of high-risk patients. The mortality rate during the studied period was 34.1%, and this was significantly associated with patient age and type of illness at presentation (p < 0.05). Stratified by year of admission, the highest rate of mortality was noted in the year 2020 (46.7%).

Conclusion

There is a high level of mortality among ICU admissions in our center. This calls for the improvement of intensive care delivery in the healthcare facility, including training and retraining of manpower and provision of essential facilities for high-quality healthcare delivery.

## Introduction

The intensive care unit (ICU) or critical care unit (CCU) is a specialized unit in a healthcare facility dedicated to the provision of care for patients who are ill from critical conditions from which there is potential for recovery [1]. The goal of the ICU is to prevent morbidity and mortality among patients who are at high risk through the provision of critical care [1]. Therefore, patients admitted to the ICU are offered more detailed observation, monitoring, and treatment as compared to the care available to patients admitted to the standard lying-in wards or departments [2].

Common conditions that are treated in ICUs include severe neurological, cardiac, and respiratory diseases [3]. A study that characterized the ICU admissions in Benue State University Teaching Hospital, Nigeria reported that the most frequent reasons for admissions were post-laparotomy care (24.8%), head injury (18.4%), and burns (11.2%) [4]. Another study conducted in southeast Nigeria among patients admitted into the ICU of the University of Nigeria Teaching Hospital, Enugu, Nigeria similarly reported postoperative cases (49.3%) as the most common reason for ICU admission, and traumatic brain injury as a leading diagnosis (70.9%) among neurosurgical patients [5]. Eze et al. examined the mortality pattern in Abakaliki, southeastern Nigeria, and revealed that acute abdomen (27.0%), trauma/injury (24.7%), and cerebrovascular accident (17.8%) were the most common reasons for admission [3]. In recent times, there has been an increasing burden of non-communicable diseases in Sub-Saharan Africa, which require ICU care [5,6].

Although mortality depends on various factors such as patient demographic, population characteristics, infrastructural availability, and type of illness, the quality of ICU care also plays a role in the clinical outcomes of patients [7]. Mortality rates in ICUs in developing countries have been remarkably higher compared to the rates in more developed countries. Studies conducted among patients in Africa reported mortality rates of 34-43% [5,6,8]. These are relatively higher than ICU mortality rates in countries like the USA (11.3%), France (18%), Australia, and New Zealand (7%) [9-11]. Evaluating the outcomes of treatment is a way to improve the quality of care, standardize conduct, and ensure effective management of resources in the ICU [12].

There has been no known published study on the diseases and mortality patterns in the ICU of Enugu State University of Science and Technology Teaching Hospital (ESUT-TH), Enugu, Nigeria. This study was therefore conducted to review the pattern of cases being admitted into the ICU of ESUT-TH as well as the clinical outcomes. The goal of this study is to elicit prospects for improvement in intensive care that would lead to better patient outcomes.

## Materials and methods

Study design

This was a three-year retrospective, descriptive review of all patients admitted into the ICU of ESUT-TH, Enugu, Nigeria from January 1, 2019, to December 31, 2021.

Study area

This study was conducted in the general ICU of ESUT-TH, Enugu, Nigeria. Enugu is in the southeastern part of Nigeria. ESUT-TH is one of the four major tertiary hospitals in Enugu State, the others being the University of Nigeria Teaching Hospital (UNTH), National Orthopedic Hospital, and Federal Neuropsychiatric Hospital. ESUT-TH has a high dependency unit (HDU), a general ICU, and a newly built isolated ICU following the coronavirus disease 2019 (COVID-19) pandemic.

The HDU is reserved for patients requiring intensive observation and care than is possible in the general wards but less than that offered in the ICU. Most patients are admitted into the HDU for close monitoring. However, patients who are at higher risk for life-threatening conditions or organ failure are moved directly to the general ICU for more intensive care. Patients admitted into the isolated ICU were excluded from this study.

The ICU of ESUT-TH has four beds equipped with ventilators, suction machines, defibrillators, i-stat machines, multiparameter monitors, and infusion and syringe pumps. The ICU of ESUT-TH is under the care of the department of anesthesia. Our ICU staff consists of six intensivists/anesthesiologists, eight intensive care nurses, eight ICU technicians, and two pharmacists. Physiotherapists, laboratory scientists, and other subspecialties are called in at any time they are needed. One consultant, one senior registrar, one registrar, and four nurses cover the ICU each day in shifts. Patients in the ICU are often discharged to the postanesthesia care unit (PACU) or general wards depending on the availability of bed space.

Data collection

Data were obtained from the admissions and discharge registers of the ICU ward. Information extracted from the registers includes age, sex, diagnosis, source of admission, duration of admission, and clinical outcome of all admissions between January 1, 2019, and December 31, 2021. Potential sources of admission include accident and emergency, lying-in wards, and operation theatres. Possible clinical outcomes include discharge to the PACU, transfer to wards, left against medical advice (LAMA), or death.

Data analysis

Data were analyzed using Statistical Package for the Social Sciences (SPSS) version 23 software (IBM Corp., Armonk, NY). Data were expressed as frequencies and percentages. Categorical parameters were compared using the chi-squared test. P-value < 0.05 was considered significant.

Ethics

Ethical approval for the study was obtained from the Ethics and Research Committee of ESUT-TH, Enugu, Nigeria (IRB number: ESUTH-P/C-MAC/RA/034/VOL.3/09). To ensure patient confidentiality, the names and any other unique identifiers were not extracted or used during data collection.

## Results

A total of 179 patients were admitted into the ICU during the three-year period. Of them, 97 (54.2%) were females. The mean age of the patients was 41.2 years. Of patients, 75 (41.9%) were young adults aged 20-39 years old, and 44 (24.6%) were middle-aged. A total of 88 (49.2%) patients admitted to the ICU were from the operating theatre, followed by 38 (21.2%) from the accident and emergency unit. Eighteen (10.1%) patients had undocumented sources of admission. The mean duration of admission was 3.8 days (Table 1).

**Table 1:** Sociodemographic characteristics of patients admitted to the ICU (n = 179)

Variable	Mean ± SD	Frequency (%)
Age (years)	41.2 ± 20.7	
0-19		23 (12.8)
20-39	-	75 (41.9)
40-59	-	44 (24.6)
60-79	-	27 (15.1)
80-99	-	10 (5.6)
Sex		
Female	-	97 (54.2)
Male	-	82 (45.8)
Year of admission		
2019	-	143 (79.9)
2020	-	30 (16.8)
2021	-	6 (3.4)
Source of admission		
Accident and emergency	-	38 (21.2)
Children emergency	-	8 (4.5)
Gynecology clinic	-	1 (0.6)
Gynecology emergency	-	1 (0.6)
Labor ward	-	4 (2.2)
Lying-in ward	-	14 (7.8)
Operating theatre	-	88 (49.2)
Referred	-	7 (3.9)
Unspecified	-	18 (10.1)
Duration of admission (days)	3.8 ± 4.5	
0-7		90 (50.3)
8-14	-	7 (3.9)
>14	-	4 (2.2)
Unspecified	-	78 (43.6)

The cases were categorized by specialties, medical or surgical, and by specific diagnoses. Categorized by specialties, this study showed that 34 (19%) admissions were general surgery cases, followed closely by 33 (18.4%) obstetrics and gynecological cases, 30 (16.8%) neurosurgical cases, and 23 (12.8%) neurological cases. There were 74 (41.3%) medical cases and 81 (45.3%) surgical cases, and the rest were unspecified. Cerebrovascular accident, which constituted 21 (11.7%) admissions, was the most common specific diagnosis, followed closely by traumatic brain injury seen in 18 (10.1%) cases (Table 2).

**Table 2:** Patterns of cases admitted to the ICU (n = 179) * Some cases had undocumented outcomes.

Variable	Frequency (%)	Mortality (%)*
Medical specialty		
General surgery	34 (19.0)	8 (23.5)
Obstetrics and gynecology	33 (18.4)	9 (27.3)
Neurosurgery	30 (16.8)	13 (43.3)
Neurology	23 (12.8)	17 (73.9)
Internal medicine	15 (9.0)	6 (40.0)
Cardiothoracic/vascular surgery	8 (4.5)	2 (25.0)
Urology	4 (2.2)	0 (0.0)
Burns and plastic surgery	2 (1.1)	0 (0.0)
Orthopedic surgery	1 (0.6)	0 (0.0)
Pediatric surgery	1 (0.6)	0 (0.0)
Psychiatry	1 (0.6)	0 (0.0)
Unspecified	26 (14.5)	-
Types of cases		
Medical	74 (41.3)	34 (45.9)
Surgical	81 (45.3)	20 (24.7)
Not documented	24 (13.4)	-
Specific diagnoses		
Stroke	21 (11.7)	17 (81.0)
Traumatic brain injury	18 (10.1)	11 (61.1)
Intestinal obstruction	11 (6.1)	4 (36.4)
Preeclampsia/eclampsia	11 (6.1)	6 (60.0)
Traumatic intracranial hemorrhage	8 (4.5)	2 (25.0)
Postpartum hemorrhage	7 (3.9)	1 (14.3)
Ruptured uterus	6 (3.4)	1 (16.7)
Sepsis	5 (2.8)	1 (20.0)
Heart failure	4 (2.2)	0 (0.0)
Thyroid disease	4 (2.2)	2 (50.0)
Renal failure	4 (2.2)	2 (66.7)
Intestinal perforation	3 (1.7)	0 (0.0)
Chest trauma	3 (1.7)	1 (33.3)
Pneumonia	2 (1.1)	1 (50.0)
Brain tumor	2 (1.1)	0 (0.0)
Fournier's gangrene	2 (1.1)	0 (0.0)
Hydrocephalus	2 (1.1)	0 (0.0)
Pericarditis/pericardial effusion	2 (1.1)	0 (0.0)
Sickle cell disease	2 (0.6)	0 (0.0)
Burns	2 (1.1)	0 (0.0)
Abruptio placenta	1 (0.6)	0 (0.0)
Asthma in pregnancy	1 (0.6)	0 (0.0)
Bladder injury	1 (0.6)	0 (0.0)
Bronchopleural fistula	1 (0.6)	1 (100)
Cardiac arrest	1 (0.6)	1 (100)
Chronic obstructive pulmonary disease	1 (0.6)	0 (0.0)
Ectopic pregnancy	1 (0.6)	0 (0.0)
Enterocutaneous fistula	1 (0.6)	0 (0.0)
Hemoperitoneum	1 (0.6)	0 (0.0)
Hypertensive encephalopathy	1 (0.6)	0 (0.0)
Liver abscess	1 (0.6)	0 (0.0)
Meningitis	1 (0.6)	0 (0.0)
Placenta previa	1 (0.6)	0 (0.0)
Prostate cancer	1 (0.6)	0 (0.0)
Rectovaginal fistula	1 (0.6)	0 (0.0)
Drug overdose in severe depression	1 (0.6)	0 (0.0)
Spine/spinal cord injury	1 (0.6)	0 (0.0)
Status epilepticus	1 (0.6)	0 (0.0)
Unspecified intestinal disease	8 (4.5)	2 (25.0)
Unspecified	6 (3.4)	-

Surveillance was the most common reason for admission to the ICU during the period, as noted in 171 (95.6%) cases, while 13 (7.3%) patients were admitted for ventilation in addition to monitoring. The reason for admission was not documented in eight (4.4%) cases. The total number of deaths recorded in the study was 61 (34.1%). Among survivors, 62 (34.6%) improved and were transferred to the lying-in wards, 14 (7.8%) were discharged home from the PACU, three (1.7%) patients left against medical advice, and one (0.6%) was referred out for further care. Of the cases, 38 (21.2%) had no documented outcomes of admission (Table 3).

**Table 3:** Reasons for and outcomes of admission to the ICU (n = 179)

Variable	Frequency (%)
Reasons for admission	
Monitoring	158 (88.3)
Monitoring and ventilation	13 (7.3)
Not specified	8 (4.4)
Outcomes of admission	
Dead	61 (34.1)
Discharged to the postanesthesia care unit	14 (7.8)
Left against medical advice	3 (1.7)
Not indicated	38 (21.2)
Referred	1 (0.6)
Transferred to lying-in ward	62 (34.6)

A chi-square analysis of factors associated with mortality among patients admitted to the ICU in the study period revealed significant associations of mortality with patient age, category of disease by specialty, and types of cases, i.e., surgical versus non-surgical, and specific diagnoses (p < 0.05; Table 4).

**Table 4:** Chi-squared analysis comparing mortality rate among patients to sociodemographic variables (p < 0.05; n = 179) * Significant.

Variable	Mortality (%)	x^2^	Df	P-value
Age (years)		11.7	4	0.02*
0-19	3 (16.7)			
20-29	20 (36.4)			
40-59	20 (52.6)			
60-79	12 (52.2)			
80-99	6 (75.0)			
Gender		2.1	1	0.18
Female	28 (37.3)			
Male	33 (49.3)			
Duration of admission (days)		0.7	2	0.79
0-7	38 (42.2)			
8-14	4 (57.1)			
>14	2 (50.0)			
Types of cases		6.9	1	0.01*
Medical	34 (45.9)			
Surgical	20 (24.7)			
Medical specialty		19.7	11	0.02*
General surgery	8 (23.5)			
Obstetrics and gynecology	9 (27.3)			
Neurosurgery	13 (43.3)			
Neurology	17 (73.9)			
Internal medicine	6 (40.0)			
Cardiothoracic/vascular surgery	2 (25.0)			

**Figure 1:**
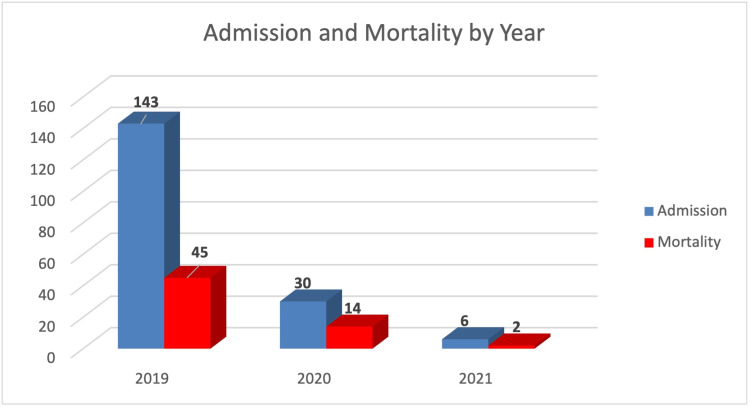
Number of patient admissions and mortalities by year of admission (n = 179)

Comparing the number of admissions and deaths by year, the number of admissions was observed to have declined from 2019 to 2021. When mortality rates were examined by year, the highest rate of mortality was seen in the year 2020 (46.7%; Figure 1).

## Discussion

The level of training and experience of staff, available resources and infrastructure, and the capacity of the ICU contribute to the outcomes of patients admitted into the ICU of any healthcare institution [13,14]. This is the first study to be carried out on the admissions and mortality pattern of cases in the ICU of ESUT-TH. A total of 179 patients were admitted over the three-year study period.

In our study, the general surgery specialty (19%) was observed to be the leading specialty utilizing ICU bed spaces. This was different from the results of a five-year retrospective study in the ICU of Lagos University Teaching Hospital, Nigeria, and a 10-year analysis in Port Harcourt, which reported the neurosurgical specialty and obstetrics and gynecology department, respectively, as the highest admitting specialties [15,16]. In our study, cerebrovascular accident (11.7%) was the most common medical diagnosis of patients admitted into the ICU while traumatic brain injury (10.1%) was the most common surgical diagnosis. This agrees with the study by Poluyi et al., which reported traumatic brain injury in 77.3% of all neurosurgical admissions and cerebrovascular accidents in 37.8% of all internal medicine admissions [15].

The overall mortality rate in the ICU during the study period was found to be 34.1%. This finding is comparable to results from other parts of Nigeria, which reported ICU mortality rates of 34.6% and 40.8% in Calabar and Abakaliki, respectively [3,17]. Other African countries reported ICU mortality rates ranging from 38.7% to 40.1% [14,18,19]. However, these are comparatively higher than the findings in more developed nations including the Scandinavian countries (Finland, Norway, and Sweden) (9.1%) and the United States of America (11.3%) [9,20]. The relatively higher mortality rate in our study may be due to the late presentation of patients in our clime, a limited number of trained ICU staff, and a dearth of adequate resources and infrastructure. Of the survivors, 7.8% of the patients were transferred to the PACU where they were managed until they were fit to be discharged to the general wards. There were no documented outcomes on 21.2% of cases, and this highlights a need for better record-keeping in our facility.

The mean age of patients in our study was 41.2 years. There was a statistically significant relationship between age groups and mortality. In general, the proportion of mortality increased with increasing age, with the highest level of mortality seen among patients aged 80-99 years old (75%) and the lowest among patients aged 0-19 years old (16.7%). This agrees with the reports of a study carried out in Ethiopia, which reported the highest rate of mortality among patients more than 60 years old [18]. However, it opposes a previous study that found no association between old age and increased mortality [3]. The reason for our results may be due to higher levels of comorbidities and more severe presentations of diseases seen among elderly patients. Although there were more female admissions than males, the proportion of death among males (49.3%) was higher than among females (37.3%). However, there was no statistically significant sex difference in mortality rates observed in this study. The mean duration of admission in our study was four days and this was similar to a previous study carried out in Abakaliki, which also documented a significant association between duration of admission and mortality [3]. However, our study showed no statistically significant relationship between the duration of admission and patient mortality.

The most common reason for admission in our study was close monitoring. In addition, 7.3% of admitted patients were admitted for mechanical ventilation. The indication for mechanical ventilation was not documented. The rate of mechanical ventilation in our study is considerably lower than the findings of a study conducted in southern Nigeria, which showed a ventilation rate of 19% among ICU admissions [17]. However, these differences might have been due to the reduced frequency of critical admissions into the general ICU following the COVID-19 pandemic as a special isolation ICU was built and used for COVID-19 cases. Admitting medical specialties, types of cases (i.e., medical/surgical), and specific diagnoses were all noted to be significantly associated with mortality rates in our study. Medical conditions had a mortality rate of 45.9% while surgical conditions had a mortality rate of 24.7%. This is similar to the findings of other studies conducted in Nigeria [3,17]. According to medical specialty, neurology (73.9%) and neurosurgery (43.3%) accounted for the highest mortality rates, followed by internal medicine (40%). A total of 100% case fatality rates were observed among patients who had a cardiac arrest and bronchopleural fistula in our study. This agrees with a multi-center cohort study, which noted that patients who sustained cardiac arrest were approximately 12 times more likely to die as compared to those who did not. This may be due to a lack of facilities for resuscitation, as well as trained staff in advanced cardiac life support (ACLS) [21].

Very high fatality rates were also noted among patients admitted with stroke (81.0%), renal failure (66.7%), intestinal obstruction (61.1%), and preeclampsia/eclampsia (60.0%) in our study. These findings are comparable to a study by Eze et al., which reported high fatality rates for stroke (71%) and renal failure (62.5%) [3].

Assessing admission and mortality trends over the study period, a sharp and progressive decline in the number of admissions was observed from 2019 (143 admissions) to 2020 (30 admissions) and 2021 (six admissions). This was due to the referral of patients suspected of having COVID-19 to isolation centers in the state. Mortality rates were also notably highest in 2020 (46.7%) compared to 31.5% and 33.3% in 2019 and 2021, respectively. This spike in death rates coincides with the peak of the COVID-19 pandemic in Nigeria, which may have caused disruptions in patient care and complicated outcomes for patients needing critical care.

Our study was limited by incomplete documentation of patient information. Some studies have reported deficiencies in the practice of documentation among nursing staff [22,23]. This may have an impact on reported patient mortality [24]. There is a need to encourage and train healthcare workers in the ICU on the need for proper documentation practices. Due to the limitations of retrospective studies, the results of this study cannot identify and account for unknown factors, which may have influenced the outcome. Therefore, we recommend the conduction of prospective studies to elicit patterns and predictors of mortality among patients getting intensive care. Multi-center reviews may also be carried out for more holistic and applicable results for the implementation of change in the country.

## Conclusions

The mortality rate noted in this study was remarkably high. This calls for the overall improvement of intensive care in the healthcare facility. There is a need for training and retraining of ICU healthcare workers, as well as the provision of important resources and facilities necessary to deliver high-quality intensive care to patients. The high level of fatalities seen among patients with cerebrovascular accidents, traumatic brain injury, renal failure, and hypertensive disorders of pregnancy (preeclampsia/eclampsia) in this study warrants the institution of public health measures that target primary prevention of these conditions, early diagnosis, and prompt treatment to improve outcomes. Considering the deficiencies in documentation, we suggest the provision of training programs to enhance the knowledge of nurses regarding documentation, adequate documentation materials, and the adoption of more reliable and efficient methods of record keeping.
